# A Brief Review on the Electrohydrodynamic Techniques Used to Build Antioxidant Delivery Systems from Natural Sources

**DOI:** 10.3390/molecules28083592

**Published:** 2023-04-20

**Authors:** Sílvia Castro Coelho, Berta Nogueiro Estevinho

**Affiliations:** 1 Laboratory for Process Engineering, Environment, Biotechnology and Energy (LEPABE), Chemical Engineering Department, Faculty of Engineering, University of Porto, Rua Dr. Roberto Frias, 4200-465 Porto, Portugal; 2 ALiCE—Associate Laboratory in Chemical Engineering, Faculty of Engineering, University of Porto, Rua Dr. Roberto Frias, 4200-465 Porto, Portugal

**Keywords:** antioxidant, delivery systems, electrospinning, electrospraying, microencapsulation, natural extract, polyphenols

## Abstract

Extracts from plants have been one of the main sources of antioxidants, namely polyphenols. The associated drawbacks, such as instability against environmental factors, low bioavailability, and loss of activity, must be considered during microencapsulation for a better application. Electrohydrodynamic processes have been investigated as promising tools to fabricate crucial vectors to minimize these limitations. The developed microstructures present high potential to encapsulate active compounds and for controlling their release. The fabricated electrospun/electrosprayed structures present different benefits when compared with structures developed by other techniques; they present a high surface-area-to-volume ratio as well as porosity, great materials handling, and scalable production—among other advantages—which make them able to be widely applied in different fields, namely in the food industry. This review presents a summary of the electrohydrodynamic processes, main studies, and their application.

## 1. Introduction

Recent consumer demands associated with health and natural issues are a focus of global interest within the scientific community [1]. Natural sources of active compounds can promote a better quality of life in different fields, such as food, nutraceuticals, pharmaceuticals, and medicine. Their properties, such as their antioxidant, antibacterial, anti-inflammatory, and antitumor activities, prevent several diseases and symptoms. The extract from plants rich in polyphenols has been one of the main sources of these natural and active compounds.

**Figure 1:**
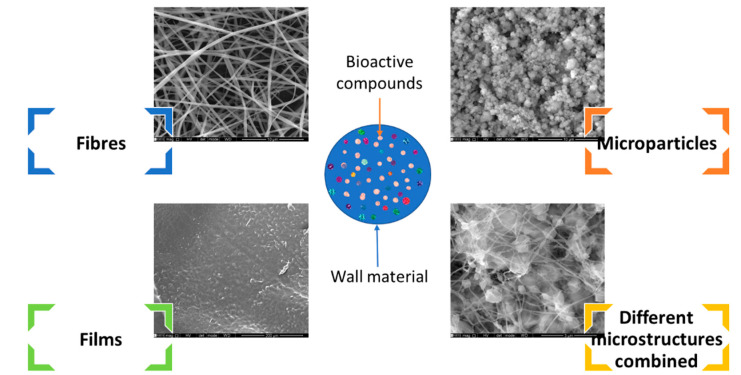
Encapsulation of bioactive compounds in different types of structures produced with electrohydrodynamic techniques. Scanning electron microscopy (SEM) images obtained by the authors with different formulations in previous works. The morphology was evaluated by scanning electron microscopy at Centro de Materiais da Universidade do Porto.

In fact, the quality and efficient improvement of these compounds’ benefits are the main strategies being considered when developing new products [2]. In this regard, microencapsulation technologies of compounds are an option to preserve and enhance the quality of the active ingredients [3,4]. These technologies involve the coating and protection of bioactive compounds by a produced barrier (wall material) (Figure 1); they allow for the improvement of the compound’s stability and solubility, thereby slowing down the release profile, and masking the flavour, odour, or taste [5].

Examples of these processes include spray drying, liposomes, spray chilling, freeze-drying, solvent evaporation, melt extrusion, liposome preparation, complex coacervation, fluidized bed coating, ionic gelation, layer-by-layer, and electrohydrodynamic techniques [6,7]. The most common technique is the spray drying [8]. In this case, the compounds are emulsified in a polymeric solution; the solution is atomized and dried in contact with hot air [9,10].

**Figure 2:**
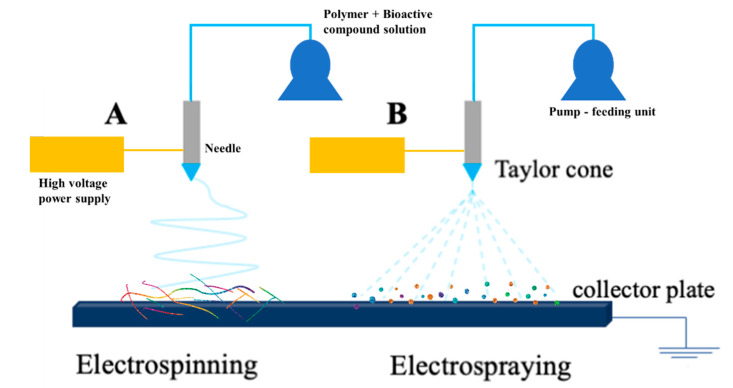
Schematic illustration of electrohydrodynamic technologies: (**A**) electrospinning, (**B**) electrospraying setups [3].

Recently, among the different microencapsulation techniques, electrohydrodynamic processes—electrospinning and electrospraying (Figure 2)—have garnered relevant consideration regarding the spray drying method [11,12]. These techniques allow for the production of structures (matrix type—the core compound is dispersed in the wall material) that are able to minimize the associated limitations of bioactive compounds, improving their stability and bioavailability [13,14].

Electrospinning is a simple, flexible, low-cost, and versatile technique of fibre production involving an electrical field, which is applied to the polymer solution from the needle to the collector [15,16]. This system is composed of a high voltage power supply, a feeding unit, and a grounded collector, as visualized in Figure 2A. The polymer is dissolved in a solvent and placed in a feeding unit. This method involves the use of high voltage electrostatic repulsive forces (Coulomb forces) to elongate and fabricate fibres [17,18,19]. When the electrical force overcomes the surface tension of the polymer, a charged jet is ejected from the Taylor cone, the fibres are formed, the solvent evaporates, and the matrix is deposited on the collector [3,17].

Electrospraying is a technique involving fabrication by using droplets in combination with electrical forces. Similar to the electrospinning process, the highly charged droplets are produced by the elongation of the polymer solution from a capillary nozzle by the Taylor cone (Figure 2B) [3,20]. The developed microparticles usually have smaller diameters (1 μm) and a low polydispersity index. As well as electrospun structures, electrospraying particles are easily produced with a low amount of associated costs. The systems are controlled through adjustments to the voltage, flow rate, and the distance to the collector. Compared with spray drying structures, electrosprayed systems have higher product yield values [9].

Electrospinning and electrospraying methods exhibit various advantages (Table 1): They are simple to use, low cost, high efficiency, and biocompounds can be easily loaded [21]. Several studies report these emergent techniques with pertinent benefits, such as the high encapsulation efficiency and an enhancement of thermosensitive biocompounds’ stability [22]. Compared with the traditional methods, an essential advantage of electrohydrodynamic techniques is the non-use of heat, thereby avoiding the loss of activity and allowing the functionality of the sensitive compounds [6,23]. On the other hand, these kinds of processes present low associated production costs when compared with traditional microencapsulation methods, such as spray drying [24]; however, all these positive characteristics enable them to be suitable methods for the design and optimization of functional nano/microsystems. The systems can be applied to food and pharmaceutical packaging to enhance the viability, controlled release, and stability of the active compounds as well as their nutraceutical properties [25].

The fabricated electrospun/electrosprayed structures present different benefits when compared with structures developed by other techniques. They present a high surface-area-to-volume ratio as well as porosity. The structures have diameters in a range of nanometres to several micrometres. For example, the electrospinning fibres have a high surface-area-to-volume ratio and porous structure, great materials handling, easy manipulation of fibre properties, and scalable production, which make them widely employed in different fields, namely in tissue engineering and regenerative medicine [25].

According to the application areas, such as food, drug delivery, pharmaceuticals, or medicine, it is possible to optimize the size and morphology of the structures. The polymer solution characteristics (type of polymer, concentration, viscosity, conductivity, surface tension, and dielectric constant), the processing parameters (flow rate, applied voltage, and distance from the needle to collector), and the external environmental conditions (temperature, humidity, oxygen, pH, and light) have a significant influence on the morphology and mechanical characteristics of the formed structures [9]. The solution characteristics are the most relevant in the microstructures’ morphology when compared with the other parameters [18]. Natural and synthetic polymers, such as whey protein, poly(ε-caprolactone) (PCL), gelatin, zein, polylactic acid (PLA), chitosan (CS), polyethylene oxide (PEO), polyvinylpyrrolidone (PVP), polyvinyl alcohol (PVA), poly(3-hydroxybutyrate-co-3-hydroxyvalerate) (PHBV), carboxymethylcellulose sodium (CMC), and their combination with different polymers were used for the production of electrospun/electrosprayed structures with low toxicity and good biocompatibility and biodegradability [17,26,27].

The viscosity, conductivity, surface charge, and tension are essential for obtaining uniform structures. Modifications in the viscosity of the polymer solution parameter (polymer chain entanglements) correspond to the stability/instability of the jet that changes the morphology of the developed structures [3]. Conductivity has an influence on the charges of the polymer jet; in fact, high conductivity allows an elongated jet and, therefore, the fabrication of uniform structures [9]. If the conductivity is too high, the jet will have a stretching response. It is essential to optimize the processing factors, thereby guaranteeing a stable Taylor cone. High voltage will allow a high amount of repulsion that leads to structures with small diameters, but excessive voltage will destabilize the Taylor cone.

This review provides an overview of the recent developments in state-of-the-art encapsulation methods that use electrohydrodynamic techniques—electrospinning and electrospraying—through the use of natural sources of active compounds, such as extracts from plants, which are rich in polyphenols and natural antioxidant compounds. Therefore, this paper begins with an introduction of the aspects of the relevant natural and active compounds, such as antioxidants and polyphenols, that are encapsulated by electrohydrodynamic processes. Likewise, the recent electrospun/electrosprayed structures that have been developed are highlighted. Several examples of natural compounds microencapsulated by electrohydrodynamic were presented and discussed. Finally, the review concludes with a summary as well as the challenges associated with the current applications of these structures.

## 2. Challenges of Microencapsulation by Electrohydrodynamic Techniques

**Figure 3:**
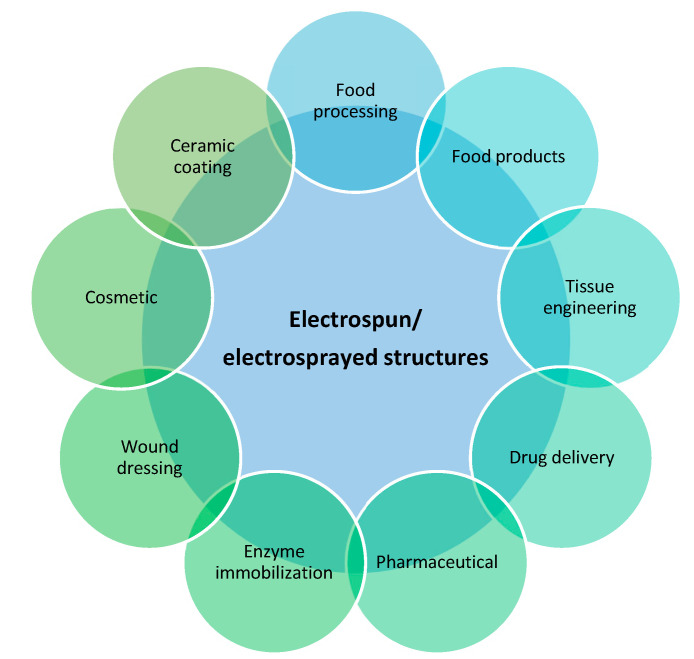
Diagram of the applications of electrohydrodynamic microstructures.

Electrospun/electroprayed structures with functional advantages can be applied to several applications, such as active food (functional products, packaging, preservation/industry), pharmaceuticals, drug delivery, enzyme immobilization, tissue engineering, and wound dressing, as observed in Figure 3 [6,28].

### 2.1. Main Applications Fields

#### 2.1.1. Food

Electrospun/electrosprayed structures with characteristics such as biocompatibility and antibacterial and antioxidant properties can be applied to the functional products, food packaging, and preservation fields [19,29,30,31]. The developed structures should maintain the food’s active compounds, which are isolated and protected from external environmental conditions, such as oxygen, odours, and moisture, which will have an impact on their activity and antimicrobial effects [17].

Therefore, functional polymeric nanofibers have emerged as promising packaging materials and remarkable breakthroughs have been made in the food packaging field [31]. As mentioned before, the electrospinning technique is recognized as a versatile and high-efficiency method to produce nanofibers with multifunctional properties and flexible structures [29,32,33]. For example, Duan et al. (2023) explored new solutions to electrospun pullulan-carboxymethyl chitosan/PEO core-shell nanofibers loaded with nanogels for food antibacterial packaging [30].

#### 2.1.2. Nutraceuticals

Nowadays, food products are not only intended to satisfy one’s hunger but also to prevent and eliminate nutrition-related diseases. In this context, the nutraceutical concept is founded. The micro/nanostructures developed by electrohydrodynamic techniques are able to be used as drug delivery systems for a dietary supplement or even prevention/treatment of diseases [34]. Nutraceutical goods allow the development of products that enhance human health and quality of life [34,35].

Given this, the term nutraceuticals itself incorporates a wide class of products that includes many categories and subcategories of compounds. One example of the application of electrodynamic methods in the food industry is in the case of prebiotics and probiotics [36]. To enhance the probiotics’ viability, Ma et al. (2023) prepared novel vehicles consisting of synthetic/natural biopolymers (polyvinyl alcohol, polyvinylpyrrolidone, whey protein concentrates, and maltodextrin), encapsulated with L. plantarum KLDS 1.0328 and gum Arabic. This prebiotic was fabricated by electrohydrodynamic techniques [36].

#### 2.1.3. Drug Delivery

Drug delivery systems can have a significant impact for medical and pharmaceutical applications [37]. Thus, they are able to target the drug in situ, thereby increasing their activity and minimizing their toxicity [38]. There are different types of compounds that can be incorporated into electrospun/electrosprayed structures to be delivered [39]. In fact, antitumor agents, antibiotics, ribonucleic acid, deoxyribonucleic acid, and proteins can be encapsulated by different biodegradable and biocompatible solutions that act as coating materials in electrohydrodynamic techniques. For example, Norouzi and Abdouss (2023) prepared electrospun nanofibers using β-cyclodextrin-grafted chitosan macromolecules loaded with indomethacin as an innovative drug delivery system [40].

#### 2.1.4. Wound Dressing

The skin is the largest organ, in terms of surface area, and protects the vital organs from the external environment. A chronic or hard-to-heal wound is also characterized by loss of residual stem cells for regeneration. The loss of residual skin stem cells for regeneration and differentiation results in the requirement of skin regeneration products to complete physiological healing [41]. Therefore, dressings play an important role in the treatment of wounds, acting both to prevent infection and accelerate healing [42]. The production of electrospun/electroprayed systems for this application has advantages compared to conventional methods [42]. An example is the inter- and intra-fibre pores and the high surface-area-to-volume that incite the reaction of fibroblastic cells and, therefore, result in better tissue formation [38,41]. Lee et al. (2023) studied the enhancement of the antibacterial activity of nanofibrous polyurethane membranes by incorporating glycyrrhizic acid-conjugated β-Cyclodextrin [43].

A specific case with regards to wounds is the wounds of diabetic people. Kumar et al. (2023) reviewed the application of polysaccharides-mediated electrospun nanofibers for diabetic wound healing [44]. Diabetic wound healing is a complex physiological process that involves a combination of various biological situations, such as hemostasis, inflammation, and remodeling. Polymeric nanofibers (electrospun fibres) offer a promising approach for the treatment of diabetic wounds and have emerged as viable options for wound management [44].

Other examples involve applications in the cosmetics field. In fact, the high surface-area-to-volume ratio of electrohydrodynamic structures improves the number of additives that can enter/exit the skin [45].

#### 2.1.5. Tissue Engineering

In regenerative medicine and bone tissue engineering, various composite materials have enormous popularity [46,47]. In terms of this, complete tissue restoration is expected, but is not always satisfactory [19,48]. Want et al. (2023) studied Chitosan/silk fibroin composite bilayer PCL (poly (ε-caprolactone)) nanofibrous mats for bone regeneration with enhanced antibacterial properties and improved osteogenic potential, which exemplified the excellent prospects of applying PCSP (combination of PCL(poly (ε-caprolactone)/CS (chitosan)-SF (silk fibroin)/PCL) mats for bone regeneration [48].

#### 2.1.6. Enzyme Immobilization

In biological processes, enzymes are normally immobilized on inert materials in order to enhance their stability and preserve their activity [38,49,50]. Some of the main challenges of enzyme application are their sensitivity to temperature, pH, and chemical conditions, as well as the excessive expenses associated with performing the enzymatic processes on a large scale (industrial plants) [51,52]. These limitations allow the development of structures that immobilize these natural biocatalysts, thereby maintaining their specificity, efficacy, and safety [27].

Another important application of enzymes is as biosensors, which represent advanced analytical devices with a fast response, as well as high selectivity and sensitivity in analyte detection. Sanz et al. (2023) prepared novel cells that integrated biosensors based on superoxide dismutase on electrospun fibre scaffolds for the electrochemical screening of cellular stress [53], and Çetin et al. (2023) prepared a compound with a highly sensitive threshold for detection of glucose via glucose oxidase immobilization on conducting polymer-coated composite polyacrylonitrile nanofibers [54].

Given this, the electrohydrodynamic techniques are being applied in numerous fields. Different projects are being developed—with interesting results—especially for food and biomedical applications. There is no doubt that electrospun and electrosprayed materials are going to have an important role in the future, namely for these types of applications. However, new studies on these subjects should be done in order to provide better solutions for existing and new problems, and to simplify the scale-up of these processes from laboratory to industrial scales.

### 2.2. Natural Biocompounds

Natural biocompounds present significant benefits for that are vital to human health. They have been investigated for pharmaceuticals, nutraceuticals, and food as well as applications related to medicine and well-being, such as tissue engineering [6]. Their properties, such as their antioxidant, antibacterial, anti-inflammatory, and antitumor activities, prevent several diseases and symptoms [55]. However, poor stability, low bioavailability, and loss of activity are inherent limits that should be considered when they are used. Microencapsulation is an attractive field that allows different tools to avoid and minimize these restrictions.

#### 2.2.1. Importance of Natural Antioxidants

Making wrong lifestyle choices can often contribute to the development of obesity, type-2 diabetes, cancer, and cardiovascular and neurodegenerative diseases [12]. An adequate diet rich in antioxidants could manage prevention at the early stages of diseases and prior to the need for therapeutic intervention.

Antioxidants are substances that may protect our cells against free radicals, which may play a role in the prevention or treatment of several diseases [56,57]. Therefore, antioxidants are compounds that have the ability to minimize oxidation [19,29]. The sources of antioxidants can be natural or artificial. In general plant-based foods are known to be rich in antioxidants [56]. Other important sources of antioxidants are vitamins. For example, Vitamin C and anthocyanins from sources such as berries and grapes are incorporated into several food products and recognized for their high antioxidant activity [11,58,59]. Another example are the carotenoids (Provitamin A, carotenoids (β-carotene, α-carotene, γ-carotene, and β-cryptoxanthin)), which are natural lipophilic pigments and antioxidants that are present in many fruits and vegetables [60]. They have a high antioxidant activity and promote free radical scavenging, which helps protect against chronic diseases [57].

However, some of these active ingredients cannot permeate into the small intestine in a sufficient enough concentration for efficacy without an efficient oral delivery system [12]. Moreover, protection during food processing and packaging can be a sensitive step that preserves the antioxidant compounds. Electrodynamic techniques are one of the options to produce active compounds with valuable therapeutic and nutraceutical applications. For example, α-tocopherol, also known as vitamin E, is a strong antioxidant that loses its activity during the packaging process [17]. Thus, the developed structures in this specific situation might be considered for the protection of vitamin E. Dumitriu et al. (2021) produced 6 μm of vitamin E-loaded PCL fibres for food packaging products with good antioxidant action [58].

Gómez-Mascaraque et al. (2019) highlighted the use of the electrospinning technique to encapsulate catechin (EGCG) and α-linoleic acid (ALA) into zein and gelatin microstructures for food-grade applications [61]. The coaxial electrospraying method allows for the enhancement of the encapsulation efficiency of the systems and, at the same time, an improvement of the EGCG and ALA protection.

Locilento et al. (2019) reported a 90% encapsulation efficiency of grape seed extract (GSE) into PLA/PEO nanofibers [62]. These biocompatible structures that were developed can be used for wound dressings. In fact, after 48 h it is observed that the GSE in the fibres will enhance the cell proliferation on fibroblast cells. The results suggested the high capacity of the system to improve viability and cell growth.

Table 2 summarizes some examples of common antioxidants (vitamins, polyphenols, carotenoids, and extracts rich in polyphenols) that have been encapsulated by electrospinning/electrospraying techniques.

#### 2.2.2. Extracts of Plants

Plants and their extracts were used in traditional medicine by different civilizations over the centuries [65,66]. Plant extracts have high concentrations of bioactive compounds in their composition [65,67] (Table 3).

The extracts of plants can be prepared by different techniques and can promote antimicrobial, antioxidant, anticancer, and anti-inflammatory activities [68,69,70]. These extracts can be obtained using different solvents, such as water, ethanol, and methanol, among others [71,72]. The solvent extraction method is the most used, but new technologies that are considered to be green are being applied [68]. For example, according to Lee et al. (2014), the ethanolic extract of yellow onion skin has 327.5 mg gallic acid equivalent per gram (GAE/g) of total phenolic and 183.95 mg quercetin equivalent per gram (QE/g) of total flavonoids.

However, several times the bioactive compounds found in the extracts of plants have low stability to processing and environmental factors, including heat, humidity, and oxygen, or chemical instability when inserted in a specific matrix [2,68,73]. Thus, they must be protected from the external environment. The microencapsulation of these extracts has been explored, namely using electrohydrodynamic techniques, as a way to protect, to maintain their bioavailability and functionality, and to facilitate the delivery of active compounds in food products [65,71,74]. For example, Vargas Romero et al. (2021) studied the effect of propolis extract encapsulation by polycaprolactone nonwovens containing chitosan that was achieved for active packaging applications, namely the protection of meat products [75]. The colour and microbial stability of fresh pork is enhanced by the propolis-PCL electrospun fibres.

Specific cases of extracts are the oleoresins and the essential oils. The oleoresins are concentrated extracts from spices, plants, and herbs that have been studied as additives in foods. The oleoresins are different from essential oils because they include volatile compounds and non-volatile compounds (pigments and pungency), making them more complex extracts, and resulting in a more complete aromatic and flavor profile. These extracts are extremely rich in compounds capable of providing aroma, taste, colour, and pungency, making oleoresins additives of interest to the food industry [68].

**Table 3:** Extracts of plants rich in antioxidant compounds, namely polyphenols and essential oils, encapsulated by electrospinning/electrospraying techniques for food and nutraceutical applications.

Extract of Plant/Antioxidant	Encapsulation Agent	Processing Parameters	Encapsulation Efficiency, %	Structures Average Size, µm	Application	Reference
Antocyanin	CS/gelatin	0.3 mL/h, 10 cm, 13 kV	40–60	micro/nanopheres	food products	[76]
Carvacrol	zein	1 mL/h, 20 cm,		0.54–0.65 fibres	active packaging	[77]
	PLA	15 kV		1.82–2.27 fibres		
	potato starch	0.6 mL/h, 20 cm, −3 and 25 cm		0.07–0.10 fibres		[78]
D-limonene	PVA	0.2 mL/h, 2 cm, 18 kV		1.75–2.84 fibers		[79]
Curcumin	PLA	15 cm, 24 kV		0.33–0.39 fibres	wound dressing	[80]
	gliadin	0.5 mL/h, 10 cm, 15 kV	81–85	0.38–0.41 fibres	food	[81]
Ferulic acid	gliadin	1 mL/h, 10 cm, 18 kV	94–97	0.27	active packaging	[82]
Quercetin	zein	1 mL/h, 10 cm, 15 kV		0.75 nanofiber	food packaging, pharmaceutical healthcare	[83]
	PCL	0.6 mL/h, 8–10 cm, 16 kV	94	0.10 fibres	wound healing	[84]
Gallic acid	lentil flour/PEO	0.6 mL/h, 30 cm, 15 kV	62.2	0.31 fibres	active packaging material	[85]
	cellulose acetate	1 mL/h; 15 cm; 15, 18, 21 kV		0.30–0.79 fibres	wound dressing	[86]
Ginger	soy protein, PEO, zein	1 mL/h, 15 cm, 24 kV		0.21–0.63	food packaging	[87]
Green tea	PVP	0.5 mL/h, 10 cm, 12.5 kV		0.34–0.39 fibres	oral products	[88]
Tea tree oil	PEO	0.6 mL/h, 15 cm, 19–25 kV	73.2	(nanofibers)	antibacterial packaging	[89]
Cinnamaldehyde essential oil	zein	0.3 mL/h, 12 cm, 13–15 kV		0.15–0.22 fibers	antibacterial package	[90]
Oregano essential oil rosemary extract green tea extract	PHBV	4 mL/h, 20 cm, 38 kV		0.80 fibres	biopackaging	[91]
Peppermint + chamomile essential oils	gelatin	0.3 mL/h, 10 cm, 15 kV		0.33–0.46 fibres	edible packaging	[92]
Thyme essential oil	gelatin	0.4 mL/h, 15 cm, 20 kV		0.21 fibers	active packaging	[93]
Propolis	Polycaprolactone (PCL) Nonwovens Containing Chitosan	50 mm/s, 18 cm, 80 kV		fibres	active packaging	[75]
Chrysin	PCL/PEG	2 mL/h, 20 cm, 18–22 kV		0.25–0.75 fibres	wound healing	[94]
Chilto	zein	0.15 mL/h, 10 cm, 11 kV	90	0.06–0.27 fibers	food packaging	[95]
Açai fruit	zein	0.4 mL/h, 10 cm, 13 kV	72.1	0.92	processed foods	[22]
Microalgal phenolic compounds	CS/PEO	300 μL/h, 10 cm, 20 kV		0.21 fibers	active packaging	[96]
Tea polyphenols	pullulan-carboxymethylcellulose sodium	0.36–0.6 mL/h, 15 cm, 19–21 kV	-	0.13 nanofibres	fruit preservation	[97]
	PLA	20 mL/h, 15 cm, 20 kV		0.49 fibres	food packaging	[23]

CS—chitosan, PLA—polylactic acid, PVA—polyvinyl alcohol, PCL—poly(ε-caprolactone), PEO—polyethylene oxide, PVP—Polyvinylpyrrolidone, PHBV—Poly(3-Hydroxybutyrate-Co-3-Hydroxyvalerate).

On the other hand, essential oils have been widely exploited for their biological properties (mainly as antimicrobials) in the food industry and pharmaceutical and biomedical fields [98]. Essential oils derived from aromatic plants have been investigated due to their nutritional and therapeutic potentials, such as their antioxidant, antimicrobial, antitumor, anti-inflammatory, and analgesic properties [42,99]. For example, lavender oil is an essential oil that can be used directly on the skin and is also commonly used in wound dressings due to its antibacterial properties and promotion of wound healing. Wang et al., (2022) used cellulose acetate and polycaprolactone as polymer carriers for silver nanoparticles and lavender oil, processing them into Janus fibres. Dede et al. (2023) used angelica root (*Angelica sylvestris*) oil encapsulated into a zein/hyaluronic acid/gelatin-based biofibre by an electrospinning process to fabricate edible food packaging materials [100]. Shao et al. (2019) reported the development of cinnamaldehyde essential oil that was incorporated into zein nanofibers by electrospinning for the active food packaging field [90]. It is verified that the combination of surfactants into zein solution allows the production of structures with small diameters. The system was investigated to preserve fresh food, wherein a decrease of microorganism growth was observed, meaning the food was in good condition.

Another study reported the potential antibacterial and antioxidant characteristics of oregano essential oil, rosemary extract, and green tea extract encapsulated into PHBV (Poly(3-Hydroxybutyrate-Co-3-Hydroxyvalerate)) electrospun fibres to be used in packaging applications [91]. A high antimicrobial effect against *E. coli* and *S. aureus* bacteria was shown. This effect was observed for oregano essential oil, which corresponded to a high inhibition that was verified by the DPPH assay.

Electrospinning has proven to be a convenient and versatile method for the encapsulation of essential oils. The electrospinning technique is able to overcome limitations of the chemical preservatives, such as low aqueous solubility and shelf life, high volatility, and activity in active food products [13].

#### 2.2.3. Polyphenols

Polyphenols are a special type of bioactive compound contained in the extracts of plants. Polyphenols are one of the main constituents of plant extracts [74]. Polyphenols are secondary metabolites that are regularly referred to as various groups of natural compounds containing phenolic functionalities, which can be found in vascular plants and present a perfect combination of biological, physiological, and chemical activities [65,74,101]. Thousands of polyphenolic compounds have been distinguished in plants and classified into various categories, depending on the number of phenol groups [2]. They are classified as flavonoids, phenolic acid, anthocyanins, and tannins, with the most significant being phenolic acids and flavonoids [2]. Flavonoids are present in fruits and vegetables that depend on the species variety, growing conditions, and maturation. They act on enzyme and vitamin synthesis. Moreover, they are essential to the colour and taste of the products’ composition. Phenolic acids are found naturally in fruits and vegetables. These compounds possess anti-inflammatory, neuroprotective, anticancer, and antidepressant activities. Anthocyanins are plant pigments that act against cancer, inflammation, and cardiovascular illnesses. Finally, tannins are compounds capable of acting in plants against UV radiation and free radical chemical signaling, as well as animal attacks [2,7,67].

The application of plant extracts rich in polyphenols has attracted pronounced interest in the nutraceutical, food, cosmetics, and pharmaceutical industries. They have been the focus of investigation due to their potential antioxidant effects against diseases and, at the same time, their ability to enhance and protect several products with regards to the food preservation and packaging, pharmaceutical, cosmetics, and chemical engineering fields [102]. They present attributes to prevent several diseases such as cancer, diabetes, and cardiovascular and neurodegenerative diseases [2,103].

Electrospinning encapsulation is a highly viable method to protect bioactive ingredients and prevent their degradation. Cruz et al. (2023) used red onion skin extract rich in flavonoids encapsulated in ultrafine fibres of sweet potato starch by electrospinning [71]. Aydin et al. (2023) used *Quercus infectoria gall-loaded* patches for wound dressing [104] and they checked that the effect of the electrospinning, 3D printing, and hydrogel casting techniques on the properties of the wound dressings was exhibited. They concluded that the highest area of the antibacterial effect belongs to the electrospinning technique [104]. Krongrawa et al. (2023) studied a *Kaempferia parviflora* extract based on electrospun shellac fibres capable of transporting methoxyflavones. The extract of this plant is used in traditional medicine to treat inflammation, swelling, wounds, bacterial infection, and ringworm, among other problems [105].

More cases of recent studies are presented in Table 3. For example, a study reported the ability of 0.1 μm quercetin-loaded PCL electrospun fibres to inhibit bacterial load and excess free radical activity in wound healing [84]. Experimental assays were performed against *S. aureus*. The non-toxicity of the nanofibers was also verified against mouse fibroblast cell lines. Aydogu et al. (2019) suggested gallic acid encapsulated into lentil flour-based electrospun fibres for active packaging applications [85]. The oxidative stability of walnuts was studied, and the results showed a reduction in walnuts’ oxidation by the use of the developed system. The development of uniform tea-loaded PLA nanofibers for food packaging is archived by Liu et al. (2018) [23]. Its antimicrobial activity against *E. coli* and *S. aureus* was examined, which verified the promising and positive results (92.26% ± 5.93% and 94.58% ± 6.53%, respectively). Another study suggested tea-loaded pullulan-CMC electrospun fibres for fruit preservation [97]. The results based on weight loss and titratable acidity demonstrated the ability of the delivery system to enhance fruit quality during storage.

Given this, electrohydrodynamic techniques are being applied to the microencapsulation of several types of antioxidant (extracts of plants, essential oils, oleoresins, or polyphenols) compounds with success. Antioxidants are natural compounds that are extremely important for our life and health and are attributed with important properties (antioxidant, antibacterial, anti-inflammatory, and antitumor activity). On the other hand, the properties of the microstructures prepared with the electrohydrodynamic techniques, such as the high surface-area-to-volume ratio (production of very thin fibres with large surface areas) are extremely important for the success of these techniques and to maintain and potentiate the biological activity of the bioactive compounds. The authors believe that electrospun fibres will have an important role in the development of new solutions for pharmaceutical and medical problems in the near future.

## 3. Relevant Physico-Chemical Analysis of Microstructures Containing Bioactive Compounds

Several physico-chemical factors might be influenced by the biocompounds’ characteristics when they are developed into the microstructures. There are several techniques that could be used to verify and characterize the systems [106,107]. Some examples are the following:

- Size, morphology/shape, and colour are the main physical properties that should be considered in the development of the polymeric microsystems. Transmission electron microscopy (TEM) and scanning electron microscopy (SEM) are methods that have been studied to observe the morphology and estimate the size of the structures. Differential scanning calorimetry (DSC) and thermogravimetric analyses (TGA) are important methods to understand the morphology of the formulated structures. In a packaging study, the behaviour of the structures can be evaluated by these techniques.
- Encapsulation efficiency is the main system characteristic to determine the compound loaded/incorporated into the microstructures. Several reports suggested the essential methods for quantifying the encapsulation efficiency: high-performance liquid chromatography (HPLC) and UV-vis spectroscopy.
- Chemical Characteristics—Fourier transform infrared spectroscopy (FTIR) is the most used technique to chemically characterize the systems and the reaction with the compounds/polymers [59,108].
- Storage Stability—The effect of environmental conditions such as temperature, oxygen, and air have an influence on the structures. For this reason, the systems must be studied in these conditions. The stability of the compounds incorporated into the electrospinning/electrosprayed structures could be evaluated and compared with the systems alone.
- Bioavailability of Biocompounds—In vitro/In vivo bioavailability of the compounds is achieved in order to simulate the compound’s effect on the system against specific conditions, such as a simulated gastrointestinal tract.
- Biological activity studies—Depending on the active compound microencapsulated, different tests can be performed to evaluate the biological activity, such as antioxidant evaluation and phenolic determination, among others [72,109,110,111].

Thus, different methods can be applied to characterize the micro/nanostructures produced by electrospinning/electrospraying techniques. These methods will be also selected by considering the type of application of the structures formed and the type of active compound incorporated.

## 4. Concluding Remarks and Future Trends

Over the past few decades, the electrohydrodynamic techniques have developed rapidly; however, some drawbacks continue to crop up, many related to the industrial scale-up.

This review covers the latest studies of improved electrospinning/electrosprayed microstructures of natural antioxidants, namely from extracts of plants and polyphenols. Antioxidants are compounds that are extremely important for our life and health that possess important properties (antioxidant, antibacterial, anti-inflammatory, and antitumor activity).

The electrohydrodynamic techniques have proven to be a convenient and versatile method for the encapsulation of antioxidants. The electrohydrodynamic processes have several advantages, thereby allowing the production of structures with natural and synthetic polymers in their composition while protecting the compounds and their properties. The fabricated electrospun/electrosprayed structures present different benefits when compared with structures developed by other techniques. They have a high surface-area-to-volume ratio as well as porosity. For example, electrospinning fibres have a high surface-area-to-volume ratio and porous structure, great materials handling, easy manipulation of fibre properties, and scalable production.

The electrohydrodynamic techniques are being applied in numerous fields, namely for food and biomedical applications. There is no doubt that electrospun and electrosprayed materials are going to have an important role in the future, namely for food and biomedical applications.

A gradual enhancement of the application of electrospinning/electrosprayed structures is expected in the next years. Future developments and processing technologies must find a way to optimize the production of electrospun/electrosprayed structures, namely in terms of industrial scale. This improvement might lead to the continuous application of the developed structures in different areas, such as food, pharmaceuticals, and biomedicine.

## Figures and Tables

**Table 1:** Advantages and disadvantages of electrohydrodynamic techniques [6,17].

Advantages	Disadvantages
Non-use of heat	Low production rate
Versatile and simple process	Expensive industrial installation
High surface-area-to-volume ratio (production of very thin fibers to the order of few nanometers with large surface areas)	Limitations in the selection of the encapsulating agents (polymers) used considering the viscosity and conductivity
High porosity	Toxicity of the residual solvent used
High encapsulation efficiency	High operating voltage
Stability of the microstructures	
Low-cost process	
Easy scale-up	
Microstructures with a wide range of applications	

**Table 2:** Examples of antioxidants encapsulated, namely vitamins, polyphenols, carotenoids, and extracts rich in polyphenols, by electrospinning/electrospraying techniques.

Antioxidant	Encapsulation Agent	Processing Parameters	Encapsulation Efficiency, %	Structures Average Size, µm	Application	Reference
Epigallocatechin gallate (EGCG)	Zein	0.15 mL/h, 10 cm, 13 kV	80	0.30 microparticles	food-grade materials	[61]
α-linolenic acid (ALA)	gelatin	0.15 mL/h, 10 cm, 18 kV	100	0.80 microparticles		
ß-carotene	whey protein	0.5 mL/h, 12 cm, 20 kV	-	0.27 nanocapsules	food packaging	[63]
α-tocopherol	PCL	0.18 mL/min, 15 cm, 15 kV		6.0 fibres		[58]
Grape seed extract	PLA/PEO	1 mL/h, 9 cm, 17 kV	90	0.30 fibres	wound dressing	[62]
Rosemary (*Rosmarinus officinalis*) polyphenols	PVA	2.2 mL/h, 20 cm, 30 kV		0.22 fibres	active packaging	[64]

PCL—poly(ε-caprolactone), PLA—polylactic acid, PEO—polyethylene oxide, PVA—polyvinyl alcohol.

## Data Availability

Not applicable.
